# Diabetic cats have decreased gut microbial diversity and a lack of butyrate producing bacteria

**DOI:** 10.1038/s41598-019-41195-0

**Published:** 2019-03-18

**Authors:** Ida Nordang Kieler, Melania Osto, Leoni Hugentobler, Lara Puetz, M. Thomas P. Gilbert, Torben Hansen, Oluf Pedersen, Claudia E. Reusch, Eric Zini, Thomas A. Lutz, Charlotte Reinhard Bjørnvad

**Affiliations:** 1 University of Copenhagen, Department of Veterinary Clinical Sciences, Dyrlaegevej 16, DK-1870 Frederiksberg C, Denmark; 2 University of Zurich, Institute of Veterinary Physiology, Winterthurerstrasse 260, CH-8057 Zurich, Switzerland; 3 University of Copenhagen, Natural History Museum of Denmark, Oester Voldgade 5-7, DK-1350 Copenhagen K, Denmark; 4 Norwegian University of Science and Technology, University Museum, 7491 Trondheim, Norway; 5 University of Copenhagen, Novo Nordisk Foundation Center for Basic Metabolic Research, Maersk Tower, Panum, Blegdamsvej 3B, DK-2200 Copenhagen N, Denmark; 6 University of Zurich, Clinic for Small Animal Internal Medicine, Winterthurerstrasse 260, CH-8057 Zurich, Switzerland; 7 University of Padova, Department of Animal Medicine, Production and Health, viale dell’Università 16, 35020 Legnaro (PD), Italy

## Abstract

Obesity and inactivity are major risk factors of feline diabetes mellitus (FDM) and human type II diabetes mellitus (T2DM). In recent years, changes in the gut microbiota have been suggested as a contributing factor to T2DM. Whether the gut microbiota (GM) composition plays a role in FDM remains unknown. The aim of the current study was firstly a cross-sectional comparison of the GM of diabetic cats, to that of lean, and of obese/overweight non-diabetic cats of a similar age. Specifically, fecal samples from 82 privately-owned cats from Denmark and Switzerland were sequenced using 16S rRNA gene amplicon metabarcoding. Secondly dietary intervention data was generated, by obtaining additional samples from a subset of cats after placing them on a high-protein diet for four weeks. The GM diversity of diabetic cats was lower than that of lean cats in the cross-sectional study, and lower compared to lean and to overweight/obese cats after diet intervention. Diabetic cats also exhibited fewer *Anaerotruncus*, *Dialister*, and unknown Ruminococcaceae than lean cats. Serum fructosamine levels correlated negatively with Prevotellaceae abundance and positively with Enterobacteriaceae abundance. In summary the intestinal microbiota of diabetic cats was characterized by decreased GM diversity and loss of butyrate producing bacterial genera.

## Introduction

Feline diabetes mellitus (FDM) shares some pathophysiological and clinical characteristics with human type 2 diabetes mellitus (T2DM), and has therefore been suggested as an animal model for T2DM^1^. Similarities with T2DM include age of onset (FDM is more common in middle aged to old cats), association with obesity, impaired insulin secretion, peripheral insulin resistance, beta cell loss and development of islet amyloid deposits in the Langerhans islets of pancreas, and complications such as polyneuropathy and retinopathy^1^. Similar to T2DM, the incidence of FDM has increased in recent years, possibly in relation to the increasing incidence of feline obesity^2^. However, despite the similarities between T2DM and FDM, FDM is often diagnosed at a late stage in which insulin therapy is necessary to achieve glycemic control and to avoid possible fatal complications^3^. Moreover, the mechanisms underlying insulin resistance and beta-cell dysfunction, ultimately leading to overt FDM are still unknown. Changes in the gastrointestinal microbial composition and function have been proposed as an additional factor that may play an important role as cause or consequence – or both – in human T2DM^4–6^.

Growing evidence indicates the presence of dysbiosis in the GM of T2DM patients^4–6^. However, inconsistencies relating to the nature of the dysbiosis exist between studies, which could possibly relate to the ethnic, dietary or therapeutic differences that have been shown to directly influence the gut microbiota^6^. In these studies, a decreased proportion of bacteria (such as *Roseburia* species and *Faecalibacterium prausnitzii*) known to produce the short chain fatty acid (SCFA) butyrate was seen in T2DM patients compared to controls^4–6^. SCFAs (butyrate, acetate, and propionate) are produced in the large intestine by certain gut microbes through fermentation of complex polysaccharides, and are known to positively influence glucose and energy metabolism^7,8^, as well as local immune function^9^. Butyrate supplies energy for the colonic epithelial cells, and may increase insulin sensitivity and energy expenditure^7^. Acetate and propionate are mostly used as substrates for lipogenesis and gluconeogenesis, respectively, in the liver. A recent study in mice found that butyrate and propionate (through different mechanisms) both activate intestinal gluconeogenesis gene expression, while propionate also acts directly as a substrate for intestinal gluconeogenesis^8^. This may seem paradoxical, as an increase in gluconeogenic substrates intuitively should be associated with decreased glycemic control. However, this does not seem to be the case with intestinal gluconeogenesis, where the released glucose is detected in the portal vein resulting in nervous signals sent to the brain leading to beneficial effects on food intake and glucose metabolism^10^. The host glucose metabolism may therefore be affected by SCFA production through intestinal gluconeogenesis.

In rodent models of obesity, gram-negative bacteria such as *Escherichia coli* have been found to increase intestinal permeability and circulating levels of bacterial lipopolysaccharide (LPS), ultimately resulting in systemic low-grade inflammation, an important feature of obesity and T2DM^11^. Higher levels of *E. coli* were also found in humans with T2DM compared to controls^4^. The low-grade systemic inflammation associated with obesity is thought to be implicated in the development of insulin resistance, and might therefore be important for the development of T2DM. This has been proposed as another mechanism through which the gut microbiota may interact with the host metabolism, leading to insulin resistance and T2DM. In agreement with findings in rats and humans, the subacute administration of LPS - which mimics low-grade inflammation in cats - impairs insulin sensitivity but not pancreatic β-cell function^12^.

Decreased gut microbial gene richness (a marker of diversity) has in humans been associated with a higher risk of obesity-related comorbidities, as determined by circulating metabolic markers^13^. However, no studies investigating the GM composition of T2D patients found decreased microbial diversity^14^.

In cats, one study found that although the microbial communities of 27 overweight/obese cats differed from 49 lean cats, no significant changes were detected in specific taxa between the two cohorts^15^. In a second study, no significant differences were found when 10 diabetic cats were compared to 20 control cats, where 10 of the controls were age/sex matched^16^. One explanation for this might be the large inter-individual differences in GM composition, and a larger sample size may be needed to document effects.

In addition to insulin treatment, a high-protein and a low-carbohydrate diet is usually recommended as a therapeutic strategy for diabetic cats^3^. There is some evidence that diabetic cats maintain hepatic gluconeogenic regulation better than diabetic humans^17^, and that a better glycemic control and an increased remission rate is achieved when cats are fed high-protein/low-carbohydrate diets^18,19^. Since the GM composition is affected by diet, it is possible that the metabolic effects exerted by the diet change when switching from a moderate-carbohydrate moderate-protein to low-carbohydrate, high-protein diet in diabetic cats, occur through alterations in the GM. In light of this, we aimed to (1) characterize the differences in the GM composition between diabetic, healthy lean, and healthy overweight/obese cats of a similar age range, and then (2) explore changes to the GM composition of a subset of these cats following one month of feeding with a high-protein/low-carbohydrate commercial diet that has been specifically developed for feline diabetes.

## Results

A total of 121 microbial DNA samples purified from feces of 82 cats were sequenced, of these, after rarefaction to 9000 reads per sample (9018–48350 reads), 23 samples had less than 9000 reads and were therefore not included in the statistical analysis. Statistical analyses were performed on the remaining samples which included 62 cats (23 DM, 15 OB, and 24 LN) from the cross-sectional study and, 36 cats (11 DM, 13 OB, and 12 LN) from the diet intervention (Supplementary Fig. S1).

To facilitate the fecal collection by the owners, cats studied were mostly living indoor, although four cats in the DM group, two cats in the LN group, and two cats in the OB group were reported to have limited outdoor access. The DM cats were either treated with Levemir (Detemir, Novo Nordisk, Bagsvaerd, Denmark, n = 11), Lantus (Glargine, Sanofi, Paris, France, n = 6), Prozinc (protamine zinc insulin, Boehringer Ingelheim, Ingelheim, Germany, n = 3) or Caninsulin (porcine insulin, MSD, Kenilworth, NJ, USA, n = 1). One of the DM cats was newly diagnosed and insulin treatment was not initiated at the time of inclusion, and for one cat, the type of insulin was unknown. For details on age, body condition score, body weight, fecal score and sex, see Supplementary Table S1. For details on complete blood count, biochemistry, thyroxine, and SAA, see Supplementary Table S2.

No significant differences were observed between Swiss and Danish cats, for any of the groups (DM, LN or OB) relating to alpha or beta diversity. Within each of the three groups (DM, OB, or LN) there were no statistically significant differences in proportions of different taxa at any taxonomic level between Swiss and Danish cats. Therefore, data from the Swiss and Danish cats were pooled for the analysis of the cross-sectional study.

### Diversity and microbial communities

#### Cross sectional study

**Figure 1:**
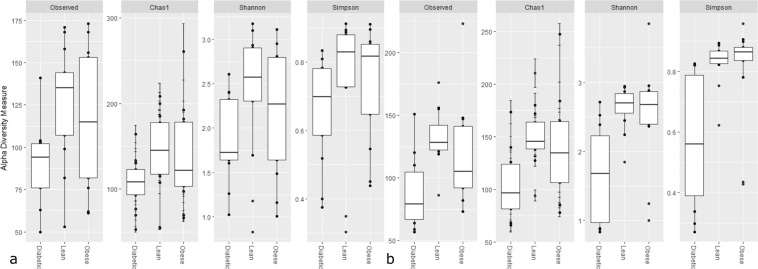
Boxplot showing the alpha diversity. Specifically the observed richness (Observed), Chao 1, Shannon diversity and Simpson diversity between diabetic (Diabetic, n = 23), overweight/obese (Obese, n = 15), and lean (Lean, n = 24), in a cross-sectional study (1A) and after a diet intervention (1B) for (Diabetic, n = 11), overweight/obese (Obese, n = 13), and lean (Lean, n = 12).

DM cats had a lower observed GM richness (false discovery rate, FDR = 0.04), and Chao 1 (FDR = 0.03) compared to the LN cats (Fig. 1A). Hemoglobin and packed cell volume level correlated positively with the observed GM richness (R^2^ = 0.48, FDR = 0.01; R^2^ = 0.41, FDR = 0.01, respectively) and Chao1 (R^2^ = 0.52, FDR = 0.01; R^2^ = 0.44, FDR = 0.01, respectively). A negative correlation was detected between observed GM richness (R^2^ = −0.44, FDR = 0.03) and Chao 1 (R^2^ = 0.44, FDR = 0.03) with serum bile acid levels. Serum fructosamine and glucose correlated negatively with the Simpson index (R^2^ = −0.23, FDR = 0.04; R^2^ = −0.32 FDR = 0.04, respectively). For further details, see Fig. S4.

#### Intervention study

After the diet intervention, the DM group retained a lower observed GM richness (FDR = 0.01) and Chao 1 (FDR = 0.03) than the LN group. In addition, lower diversity was seen for the DM cats than either the LN (Shannon index, FDR = 0.003, Simpson index, FDR = 0.002) or the OB groups (Shannon index, FDR = 0.01, Simpson index, FDR = 0.003, Fig. 1B).

A small separation of microbial communities among the three groups was detected in both the cross sectional and intervention study, independent of the dissimilarity measure used (Bray Curtis (Bray), unweighted or weighted UniFrac), (Bray cross-sectional study, R = 0.08 and FDR = 0.01; Bray intervention study, R = 0.17 and FDR = 0.002; unweighted cross-sectional study, R = 0.10 and FDR = 0.001; unweighted intervention study, R = 0.11 and FDR = 0.02; weighted cross-sectional study R = 0.11 and FDR = 0.004; weighted intervention study, R = 0.11 and FDR = 0.02). Results from the pair-wise comparisons of each group (DM, LN, and OB) for the cross sectional as well as the intervention study are depicted in Table 1.

**Table 1:** Pair-wise comparison (analysis of similarity, ANOSIM) of the microbial communities of the cats in the cross-sectional diabetic (DM), (n = 23), lean (LN), (n = 24) and overweight/obese (OB) (n = 15), as well as the diet-intervention study (DM, n = 11; LN, n = 12; OB, n = 13) with three different dissimilarity measures; Bray Curtis, unweighted and weighted UniFrac.

		Dissimilarity	ANOSIM (R)	FDR
Cross-Sectional	DM vs. LN	Bray Curtis	0.018	0.2
	DM vs. LN	unweighted UniFrac	**0.2**	**0.003**
	DM vs. LN	weighted UniFrac	0.021	0.2
	DM vs. OB	Bray Curtis	**0.084**	**0.03**
	DM vs. OB	unweighted UniFrac	**0.17**	**0.008**
	DM vs. OB	weighted UniFrac	**0.12**	**0.03**
	OB vs. LN	Bray Curtis	0.03	0.2
	OB vs. LN	unweighted UniFrac	0.09	0.05
	OB vs. LN	weighted UniFrac	0.03	0.2
Diet-intervention	DM vs. LN	Bray Curtis	**0.17**	**0.01**
	DM vs. LN	unweighted UniFrac	**0.29**	**0.006**
	DM vs. LN	weighted UniFrac	**0.16**	**0.02**
	DM vs. OB	Bray Curtis	**0.19**	**0.02**
	DM vs. OB	unweighted UniFrac	**0.14**	**0.03**
	DM vs. OB	weighted UniFrac	0.1	0.06
	OB vs. LN	Bray Curtis	0.02	0.3
	OB vs. LN	unweighted UniFrac	−0.03	0.7
	OB vs. LN	weighted UniFrac	−0.02	0.6

There was no effect of breed, sex, or age on the microbial communities. To elucidate the genera most important for the beta diversity, the most common genera were fitted as vectors for the first and second principal coordinates of the Bray Curtis dissimilarity, and genera found to have a significant effect on the variance were plotted to illustrate their effect on the variation (see Supplementary Fig. S2).

### Differences in microbiota composition between groups in the cross sectional and diet intervention studies

**Figure 2:**
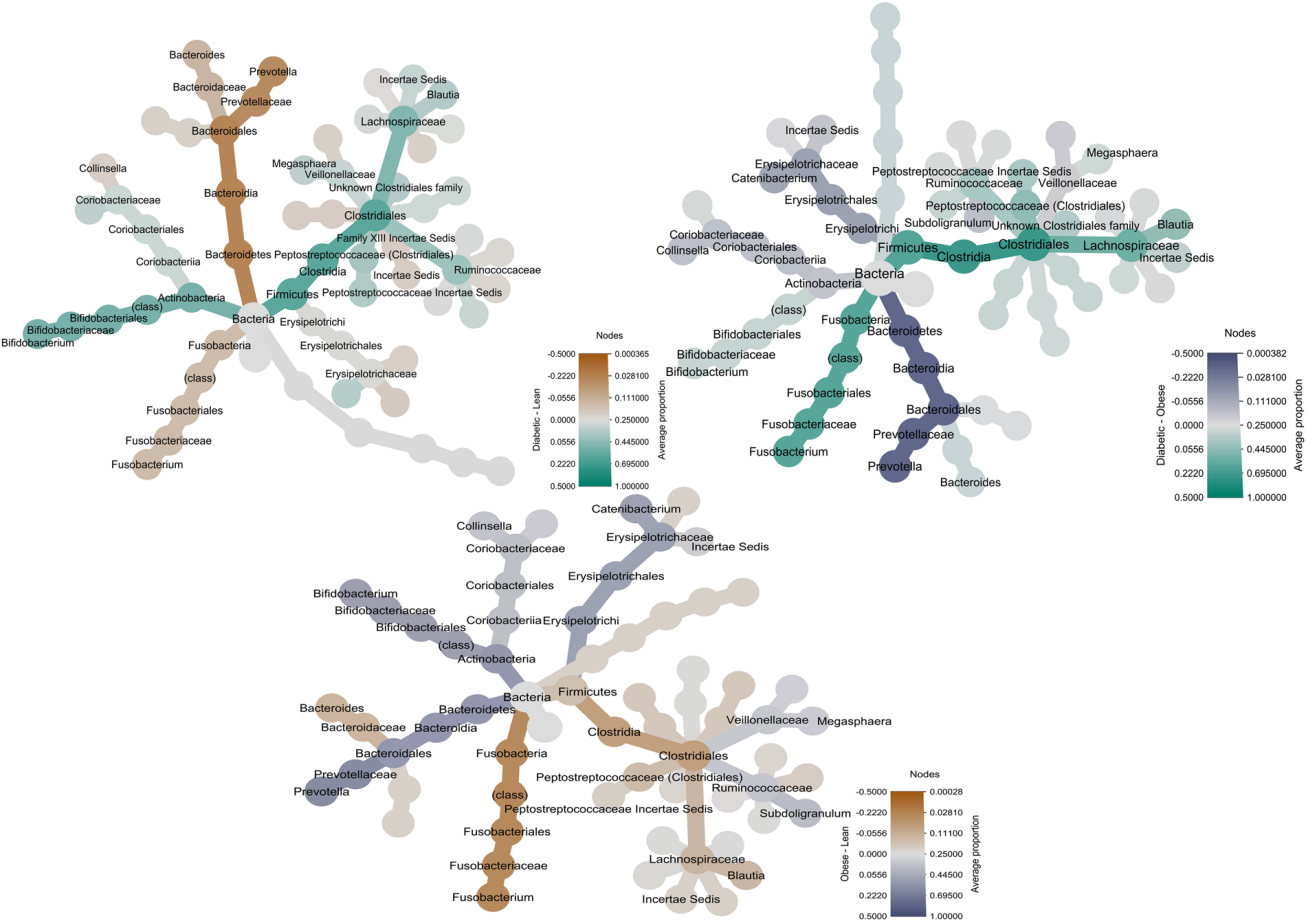
Phylogenetic tree giving an overview of the differences in abundance of gut microbiota between groups. Showing the proportions of bacteria from phylum to genera between Diabetic (n = 23), Lean cats (n = 24) as well as between Overweight/obese (Obese) (n = 15) cats in the cross sectional study.

There were multiple specific differences in the proportions of the relative abundance of various taxa from phylum to genus level when comparing the DM, OB and LN cats (see Fig. 2). Therefore, only selected differences are mentioned here while the results of this analysis, including effect size of the differences and the standard error of the effect size in their entity are shown in Table 2. When comparing the DM cats with the LN and OB cats, the DM cats had a deceased proportion of Bacteroidetes, Bacteroida, Bacteroidales, Prevotellaceae, and *Prevotella*. Similarly the DM cats had a decreased proportion of the genus *Anaerotruncus*, of the phylum Firmicutes. Compared to the DM cats the LN cats had a larger proportion of the Fusobacteria phylum and class, Fusobacteriales and Fusobacteriaceae. While the OB cats compared to the DM cats had a decreased proportion of the Fusobacteria phylum and class, Fusobacteriales and Fusobacteriaceae and *Fusobacterium*. The OB cats had a larger proportion of Actinobacteria phylum and class, Bifidobacteriales, Bifodobacteriaceae, and *Bifidobacterium* compared to the LN cats. Following the 4 week diet intervention, the only significant difference between groups at taxonomic levels above genera was a significantly decreased proportion of the Coriobacteriia class and the Coriobacteriales order in the OB compared to the LN cats. DM cats had a decreased proportion of the *Dialister* genus, and an increased proportion of an unknown *Lachnospiraceae* genus compared to the LN cats. The *Dialister* genus was also decreased in the DM cats compared to the OB, while the *Peptostreptococcaceae Incertae Sedis* genus was increased in the DM compared to the OB cats. Further details are included in Table 3.

**Table 2:** Differences in rarefied relative abundance of the most common gut microbiota at phylum to genus level, between diabetic (DM), (n = 23), lean (LN), (n = 24) cats as well as overweight/obese (OB), (n = 15) cats. Differences are illustrated as effect size (E) expressed as the difference between groups in terms of the binary logarithm (2^n^), including the standard error (SE) of the effect size and the false discovery rate (FDR) adjusted p-value, which was considered significant at FDR < 0.05, (bold).

	DM vs. LN			DM vs. OB			OB vs. LN		
	E	SE	FDR	E	SE	FDR	E	SE	FDR
**Phylum**									
Actinobacteria	1.7	0.9	0.09	−1.5	1.0	0.2	**3.2**	1	**0.004**
Bacteroidetes	−**4.6**	**0.8**	**1.8E-07**	−**6.8**	**0.9**	**2.2E-12**	**2.2**	**0.9**	**0.02**
Firmicutes	0	0	1	**3.5**	**1.1**	**0.004**	0	0	1
Fusobacteria	−**3.8**	**1**	**3.0E-10**	−9.8E-10	0.01	1.0	−**7.2**	**1.1**	**6.4E-10**
**Class**									
Erysipelotrichi	−1.2	0.8	0.2	−**3.0**	**0.9**	**0.002**	1.8	0.9	0.06
Coriobacteriia	−0.2	0.9	1	−**3.6**	**1.0**	**0.002**	**3.4**	**1**	**0.002**
Actinobacteria (class)	**7.4**	**1.2**	**1.9E-09**	−0.3	1.3	1.0	**7.6**	**1.3**	**1.8E-08**
Bacteroidia	−**4.6**	**0.8**	**1.3E-07**	−**6.8**	**0.9**	**2.7E-12**	**2.2**	**0.9**	**0.03**
Fusobacteria (class)	−**3.7**	**1**	**5.0 E-04**	**3.5**	**1.1**	**0.004**	−**7.2**	**1.1**	**2.5E-09**
Clostridia	0	0	1	−9.2E-10	0.01	1.0	0	0	1
**Order**									
Erysipelotrichales	−1.2	0.8	0.2	−3.0	0.9	0.0	1.8	0.9	0.06
Coriobacteriales	−0.2	0.9	1	−3.6	1.0	0.0	**3.4**	**1**	**0.002**
Bifidobacteriales	**7.4**	**1.2**	**1.9E-09**	−0.3	1.3	1.0	**7.6**	**1.3**	**1.8E-08**
Bacteroidales	−**4.6**	**0.8**	**1.3E-07**	−**6.8**	**0.9**	**2.7E-12**	**2.2**	**0.9**	**0.03**
Fusobacteriales	−**3.7**	**1**	**5.0E-04**	**3.5**	**1.1**	**0.004**	−**7.2**	**1.1**	**2.5E-09**
Clostridiales	0	0	1	0.0	0.0	1.0	0	0	1
**Family**									
Family XIII Incertae Sedis	−1.4	0.8	0.2	−0.5	1.0	0.7	−0.9	1	0.5
Peptococcaceae	−0.8	0.7	0.4	0.02	0.8	1.0	−0.8	0.8	0.5
Clostridiaceae	−1.8	1.2	0.2	−1.2	1.4	0.6	−0.6	1.4	0.8
Veillonellaceae	−2.2	1.2	0.2	−**5.3**	**1.3**	**3.0 E-04**	3.2	1.3	0.05
Erysipelotrichaceae	−0.3	0.8	0.8	−**3.1**	**0.9**	**0.002**	**2.8**	**0.9**	**0.007**
Coriobacteriaceae	−0.2	0.9	0.8	−1.8	1.0	0.2	1.7	1	0.2
Bifidobacteriaceae	**8.3**	**1.2**	**4.4E-11**	−0.7	1.3	0.7	**9.0**	**1.3**	**2.2E-10**
Bacteroidaceae	−**1.9**	**0.7**	**0.04**	−0.8	0.8	0.6	−1.1	0.8	0.3
Prevotellaceae	−**5.6**	**1**	**10.0E-08**	−7.5	1.1	0.0	1.9	1.1	0.2
Porphyromonadaceae	−1.4	1	0.2	0.2	1.1	0.9	−1.6	1.1	0.2
Ruminococcaceae	0.9	0.5	0.2	−0.7	0.6	0.5	**1.6**	**0.6**	**0.03**
Unknown Erysipelotrichales	−1.2	1.1	0.4	−**3.5**	**1.2**	**0.01**	2.2	1.2	0.1
Fusobacteriaceae	−**2.9**	**1**	**0.02**	**3.3**	**1.1**	**0.01**	−**6.2**	**1.1**	**3.9E-07**
Unknown Clostridiales	0.5	0.4	0.4	0.6	0.5	0.5	0	0.5	1
Peptostreptococcaceae (Clostridiales)	0.2	0.5	0.8	0.2	0.5	0.8	0	0.5	1
Lachnospiraceae	−0.1	0.3	0.8	−0.3	0.4	0.6	0.2	0.4	0.7
**Genus**									
*Roseburia*	−1.1	1	0.5	0.3	1.2	0.9	−1.4	1.2	0.4
*Lachnospira*	1.3	1.2	0.5	−1.1	1.3	0.6	2.4	1.3	0.2
*Family XIII Incertae Sedis*	0.2	0.9	0.9	1.2	1.0	0.5	−1	1	0.5
*Lachnospiraceae Incertae Sedis*	−8E-09	0	1	−8E-09	0.1	1.0	2.0E-16	0.1	1
*Peptococcus*	−1	0.7	0.4	−0.2	0.8	0.9	−0.7	0.8	0.5
*Clostridium (Clostridiaceae)*	−1.9	1.2	0.4	0.3	1.4	0.9	−2.1	1.4	0.3
*Megasphaera*	−2.7	1.2	0.1	−**5.9**	**1.3**	**6.9E-05**	**3.2**	**1.3**	**0.04**
*Dialister*	1.3	1.5	0.6	−3.2	1.7	0.2	**4.5**	**1.7**	**0.04**
*Peptostreptococcaceae Incertae Sedis*	0.9	0.7	0.4	0.3	0.8	0.8	0.6	0.8	0.6
*Catenibacterium*	0.7	1	0.6	−**5.0**	**1.1**	**6.9E-05**	**5.7**	**1.1**	**3.9E-06**
*Erysipelotrichaceae Incertae Sedis*	−0.9	0.8	0.5	−2.1	0.9	0.1	1.2	0.9	0.4
*Solobacterium*	−1.9	1.5	0.4	−1.8	1.6	0.5	−0.1	1.6	1
*Collinsella*	−0.1	1	1	−0.5	1.1	0.8	0.4	1.1	0.8
*Unknown Coriobacteriaceae*	**3.5**	**1.1**	**0.01**	1.1	1.2	0.6	2.4	1.2	0.1
*Bifidobacterium*	**8**	**1.2**	**1.4E-10**	1.2	1.3	0.5	**6.7**	**1.3**	**2.3E-06**
*Bacteroides*	−1.4	0.8	0.3	0.9	0.9	0.5	−**2.3**	**0.9**	**0.04**
*Prevotella*	−**4.7**	**1**	**3.6E-05**	**−6.5**	**1.1**	**1.2E-07**	1.9	1.1	0.2
*Parabacteroides*	−1.8	0.9	0.2	1.2	1.1	0.5	−**3**	**1.1**	**0.03**
*Subdoligranulum*	0.9	0.9	0.5	−**2.7**	**1**	**0.04**	**3.6**	**1**	**0.003**
*Faecalibacterium*	−0.7	0.8	0.6	0.3	0.9	0.8	−1	0.9	0.4
*Anaerotruncus*	−**2.8**	**0.8**	**0.01**	−**2.7**	**0.9**	**0.02**	−0.1	0.9	1
*Fusobacterium*	−1.6	1.1	0.4	**5.5**	**1.2**	**5.2E-05**	−**7.1**	**1.2**	**8.1E-08**
Unknown Lachnospiraceae	0.3	0.6	0.7	0.4	0.7	0.8	−0.1	0.7	1
Unknown Ruminococcaceae	0.6	0.9	0.7	1.4	1.1	0.5	−0.9	1.1	0.6
*Coprococcus*	0.5	0.6	0.6	1.0	0.7	0.4	−0.5	0.7	0.6
*Blautia*	0	0.5	1	0.7	0.5	0.5	−0.6	0.5	0.4

**Table 3:** Differences in rarefied relative abundance of the most common gut microbiota at phylum to genus level, between diabetic (DM), (n = 11) and lean (LN), (n = 12) cats as well as overweight/obese (OB), (n = 13) cats following dietary intervention. Differences are illustrated as effect size expressed as the difference between groups in terms of the binary logarithm (2^n^), including the standard error (SE) of the effect size (E) and the false discovery rate (FDR) adjusted p-value, which was considered significant at FDR < 0.05, (bold).

	DM vs. LN			DM vs. OB			OB vs. LN		
	E	SE	FDR	E	SE	FDR	E	SE	FDR
**Phylum**									
Actinobacteria	−1.3	1.3	0.6	0.2	1.3	1.0	−1.5	1.3	0.3
Bacteroidetes	−1.5	1.2	0.6	−**3.4**	**1.2**	**0.02**	1.9	1.2	0.3
Fusobacteria	0.8	1.5	0.8	−1.1	1.4	0.9	1.9	1.4	0.3
Firmicutes	0	0	1	−8E-15	0.02	1.0	0	0	1
**Class**									
Erysipelotrichi	−0.5	1.2	0.8	−0.6	1.2	0.7	0.1	1.2	1
Coriobacteriia	−1.4	1.4	0.8	2.5	1.3	0.2	−**3.8**	**1.3**	**0.02**
Actinobacteria	−1.1	1.7	0.8	−0.8	1.7	0.7	−0.3	1.6	1
Bacteroidia	−1.5	1.2	0.8	−3.4	1.2	0.0	1.9	1.2	0.3
Fusobacteria	0.8	1.5	0.8	−1.1	1.5	0.7	1.8	1.4	0.4
Clostridia	−4E-15	0.02	1	−3E-15	0.02	1.0	−1E-15	0.02	1
**Order**									
Erysipelotrichales	−0.5	1.2	0.8	−0.6	1.2	0.7	0.1	1.2	1
Coriobacteriales	−1.4	1.4	0.8	2.5	1.3	0.2	−**3.8**	**1.3**	**0.02**
Bifidobacteriales	−1.1	1.7	0.8	−0.8	1.7	0.7	−0.3	1.6	1
Bacteroidales	−1.5	1.2	0.8	−3.4	1.2	0.0	1.9	1.2	0.3
Fusobacteriales	0.8	1.5	0.8	−1.1	1.5	0.7	1.8	1.4	0.4
Clostridiales	−4E-15	0.02	1	−3E-15	0.02	1.0	−1E-15	0.02	1
**Family**									
Family XIII Incertae Sedis	−1.3	1.3	0.6	0.7	1.2	0.8	−2.0	1.2	0.3
Peptococcaceae	−0.2	1.1	0.9	0.2	1.0	0.9	−0.4	1.0	0.8
Clostridiaceae	−2.9	1.9	0.6	−**5.6**	**1.8**	**0.01**	2.7	1.7	0.3
Veillonellaceae	−1.5	1.7	0.7	−3.2	1.7	0.1	1.8	1.6	0.5
Erysipelotrichaceae	−0.3	1.2	0.9	−0.3	1.1	0.9	0.03	1.1	1.0
Coriobacteriaceae	−2.4	1.3	0.6	1.8	1.3	0.3	−**4.2**	**1.3**	**0.01**
Bifidobacteriaceae	−0.1	1.8	1.0	−0.1	1.7	0.9	0.1	1.6	1.0
Bacteroidaceae	−1.2	1.1	0.6	−2.1	1.0	0.1	0.9	1.0	0.6
Prevotellaceae	−0.3	1.4	0.9	0.2	1.4	0.9	−0.5	1.4	0.8
Porphyromonadaceae	−0.4	1.5	0.9	−1.3	1.4	0.6	0.9	1.3	0.7
Ruminococcaceae	−0.2	0.8	0.9	−1.7	0.8	0.1	1.4	0.8	0.2
Unknown Erysipelotrichales	−1.8	1.5	0.6	−1.0	1.5	0.8	−0.9	1.4	0.7
Fusobacteriaceae	1.6	1.5	0.6	−0.1	1.4	0.9	1.7	1.4	0.4
Unknown Clostridiales	0.2	0.7	0.9	1.4	0.6	0.1	−1.2	0.6	0.2
Peptostreptococcaceae (Clostridiales)	1.9	0.7	0.1	**2.9**	**0.6**	**5.0E-05**	−1.0	0.6	0.3
Lachnospiraceae	−0.6	0.5	0.6	−**2.2**	**0.5**	**5.0E-05**	**1.5**	**0.5**	**0.01**
**Genus**									
*Roseburia*	−1.5	1.6	0.7	−4.1	1.6	0.1	2.6	1.4	0.4
*Lachnospira*	−1.3	1.7	0.8	−3.7	1.6	0.1	2.3	1.5	0.5
*Family XIII Incertae Sedis*	−0.3	1.4	1	1.3	1.3	0.6	−1.6	1.3	0.6
*Lachnospiraceae Incertae Sedis*	0	0.1	1	0.0	0.1	1	0	0.1	1
*Peptococcus*	1.2	1.1	0.6	1.3	1.1	0.5	−0.1	1	1
*Clostridium (Clostridiaceae)*	−3	1.9	0.4	−4.6	1.8	0.1	1.6	1.7	0.6
*Megasphaera*	0.6	1.7	1	−0.9	1.7	0.9	1.5	1.6	0.6
*Dialister*	−**6.8**	**2.3**	**0.04**	−**10.8**	**2.2**	**3.0E-05**	4	2.1	0.4
*Peptostreptococcaceae Incertae Sedis*	1.6	1	0.4	**3.3**	**1.0**	**0.008**	−1.7	1	0.4
*Catenibacterium*	0.4	1.5	1	0.6	1.4	0.9	−0.3	1.4	1
*Erysipelotrichaceae Incertae Sedis*	−1.6	1.2	0.5	−1.1	1.2	0.6	−0.5	1.2	0.9
*Solobacterium*	−0.2	2.1	1	0.5	2.1	0.9	−0.8	2	0.9
*Collinsella*	−1	1.5	0.8	3.0	1.4	0.1	−4	1.4	0.1
Unknown Coriobacteriaceae	0.6	1.7	1	2.4	1.6	0.4	−1.8	1.6	0.6
*Bifidobacterium*	0.1	1.7	1	0.7	1.7	0.9	−0.6	1.6	0.9
*Bacteroides*	0.5	1.2	1	0.4	1.1	0.9	0.2	1.1	1
*Prevotella*	−1.5	1.5	0.6	−1.4	1.4	0.6	−0.2	1.4	1
*Parabacteroides*	0.4	1.4	1	−0.2	1.4	1.0	0.5	1.3	0.9
*Subdoligranulum*	−2.7	1.3	0.2	**−4.1**	**1.3**	**0.0** **1**	1.5	1.2	0.6
*Faecalibacterium*	1.8	1.2	0.4	−0.2	1.1	1.0	2.1	1.1	0.4
*Anaerotruncus*	2.4	1.2	0.2	1.2	1.1	0.6	1.1	1.1	0.6
*Fusobacterium*	0.1	1.6	1	−1.2	1.5	0.7	1.3	1.5	0.6
Unknown Lachnospiraceae	**2.7**	**0.9**	**0.04**	1.7	0.8	0.1	1	0.8	0.6
Unknown Ruminococcaceae	3.7	1.4	0.1	1.8	1.3	0.4	1.9	1.3	0.5
*Coprococcus*	−0.6	0.8	0.8	−0.1	0.8	1.0	−0.5	0.8	0.8
*Blautia*	−0.9	0.7	0.5	−0.5	0.7	0.7	−0.4	0.7	0.8

### Correlation analyses and Canonical Correspondence Analysis (CCA)

**Figure 3:**
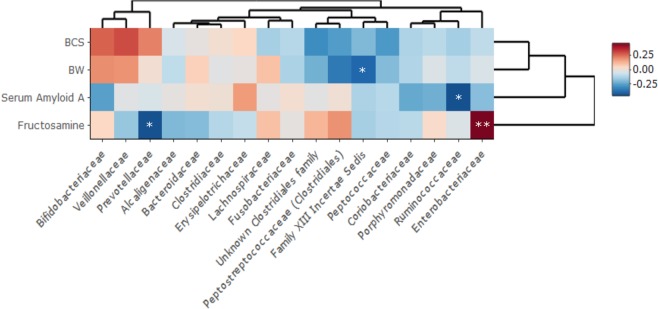
Heatmap of the correlation between the relative abundance of the gut microbiota and specific clinical parameters. Pairwise Spearman correlation of the most abundant family level relative abundance microbiota data and the body weight, body condition score, Serum Amyloid A (SAA), and fructosamine levels of all the cats (diabetic (n = 23), lean (n = 24), and overweight/obese (n = 15)) from the cross-sectional study was performed. P-values were adjusted using FDR and were considered significant when FDR < 0.05. *FDR < 0.05, **FDR < 0.01.

Correlating the most abundant microbiota data at family level with the body condition score, the body weight (BW), and the serum levels of fructosamine and SAA revealed significant correlations with several gut microbial families (see Fig. 3 for details). Serum fructosamine levels correlated positively with Enterobacteriaceae (R^2^ = 0.47, FDR < 0.01), while there was a negative correlation with the Prevotellaceae (R^2^ = −0.43, FDR < 0.05). SAA levels correlated negatively with the Ruminococcaceae relative abundance (R^2^ = −0.43, FDR < 0.05). Further, body weight showed a negative correlation with Family XIII Incertae Sedis (R^2^ = −0.38, FDR < 0.05).

At the OTU level, 83% of the variation in the GM was explained by complete blood count, biochemistry values, group (LN, OB, and DM), country, body weight, body condition score, age, sex, neutering, and fecal score (Supplementary Fig. S3). Of these variables tested, ten had a significant effect on the microbial variation at the OTU level after FDR adjustments, these included belonging to the LN or OB group, hemoglobin levels and serum levels of creatinine, bilirubin, alkaline phosphatase, magnesium, phosphorous, glucose and fructosamine (Supplementary Fig. S3).

### Predicted functional profiles

The average nearest sequenced taxon index (NSTI) and standard deviation was 0.07 ± 0.02. Low NSTI scores close to 0.06 indicate that PICRUST was able to predict the functions of the feline gut microbes. For further details see Supplementary Table S3.

## Discussion

In this study of cats sampled in two different European countries, we demonstrated that diabetic cats exhibited an overall significantly reduced richness of the GM compared to lean healthy cats. Following a 4-week dietary intervention with a high-protein commercial diet formulated for diabetic cats, the DM cats exhibited decreased richness and evenness compared to LN cats, the microbial GM community of DM cats differed from that of LN and OB cats, and a decreased evenness was also seen in DM cats compared to OB cats. The cross sectional study also revealed differences in the microbial communities (expressed as beta-diversity) of the DM, compared to LN, and OB cats. Interestingly, no significant difference was seen in the microbial community between LN and OB cats, whether in the cross sectional or intervention study. Correlation was also detected between a decreasing diversity, serum fructosamine, and circulating levels of glucose and bile acids levels in the cross sectional study. The correlation with fructosamine and glucose is not surprising considering the differences in richness and diversity between the DM cats and the LN cats. The correlation between serum bile acid levels and decreasing gut microbial diversity is also of interest, considering that the production of secondary bile acids by the GM has been suggested as a mechanism by which GM affect the host glucose metabolism^20^. Increasing diversity on the other hand correlated with hemoglobin and packed cell volume in the cross sectional study, and a correlation between GM composition and hemoglobin has also been noted in humans where a connection with more oxygen dependent microbes was suggested^21^. It is possible that the cats with a more diverse GM also had higher levels of more oxygen dependent bacteria^22^.

The decreased richness and diversity of the DM cats was further reflected in the finding that almost all differences observed between diabetic and lean cats independent of taxonomic level resulted from a decrease in the specific taxa in the GM of DM cats. Differences in the microbiota composition between DM and healthy LN and OB cats were reflected in differences in predicted metabolic functions of the gut bacteria. After feeding a diabetic diet to a proportion of the cats for four weeks, fewer differences were seen between the microbial taxa of diabetic compared to lean as well as between OB and LN cats. The reason for a more similar GM in the three groups following intervention could be a result of decreasing the confounding dietary effects, but also, a small sample size could contribute to the lack of significant differences.

Decreased gut microbial diversity is a common feature of several human diseases^23–25^. Decreased diversity, as a feature of dysbiosis, has been described in dogs suffering from several acute and chronic diseases, such as exocrine pancreatic insufficiency^26^, acute diarrhea^27^, idiopathic inflammatory bowel disease^28^ and obesity^29^. In cats, decreased bacterial diversity has been associated with acute and chronic diarrhea^30^. However, decreased microbial diversity has neither been reported as a feature of the gut microbiota of human T2DM^14^, nor reported in a previous study comparing the GM composition of diabetic with healthy cats^16^. Interestingly, in this study, the difference in diversity between DM and healthy LN and OB cats actually increased following the dietary intervention (see Fig. 1B).

In the human gut, the proportion of Fusobacteria (and specifically *F.nucleatum*) increases during colorectal cancer^31^. These bacteria are typically more prevalent in carnivores (including avian^32^, reptilian, marine mammal^33,34^, dogs and cats^35^) than in humans. Currently there is a lack of knowledge regarding the roles of Fusobacteria in the microbiota of carnivores. In vultures, Fusobacterium appears to be harmless or even beneficial for the host^32^, and it is therefore possible that the same is true in cats and dogs. In our study, there was a decreased proportion of the Fusobacteria and the Fusobacteriaceae in DM cats, but no significant change was seen in any *Fusobacterium* genera. We speculate that the decreased proportion of Fusobacteria in DM cats compared to LN may simply be the consequence of the reduced bacterial diversity seen in the diabetic group. However, the implication of the decreases of Fusobacteria in DM and OB cats as compared to LN cats is currently unknown, and the relation to decreased diversity does not explain the lower proportion in the OB cats.

In the DM cats the decreased proportion of the *Prevotella* genus may be particularly interesting since a negative correlation was seen between the Prevotellaceae family and serum fructosamine levels. Serum fructosamine is used to monitor and control blood glucose and to diagnose diabetes in cats since it is a marker of long term (2–3 weeks) blood glucose levels, similarly to hemoglobin A1c (HbA1c) in humans^36^. Interestingly, a 3-day barley kernel diet intervention in healthy humans, improved glucose and insulin responses, but only when a concomitant increase in the abundance of *Prevotella copri* was seen^37^. Furthermore, the same study reported that germ-free mice showed improved glucose tolerance and increased hepatic glycogen storage than control mice when they received daily gavage with live *P. copri* isolated from human feces^37^. In general carbohydrate and fiber rich diets are associated with increases in the *Prevotella* genus in the gut. Similarly, a study in growing kittens revealed that a moderate protein and moderate carbohydrate diet led to an increased proportion of *Prevotella*, while a high protein and low carbohydrate diet, resulted in increased *Bacteroides*^38^. This could indicate that also in cats, *Prevotella* levels are correlated with dietary carbohydrates while *Bacteroides* is correlated with animal protein. However, the presence of the *Prevotella* genus in the GM of humans has also been associated with disease^39,40^.

The *Prevotella* genus has large genetic variability between different species and strains^41^. This may be an explanation for these conflicting results regarding whether the presence of *Prevotella* in the gut should simply viewed as a reflection of dietary composition, and/or if its presence is beneficial or harmful to the host. Understanding the function of different *Prevotella* species and strains through full metagenomic characterizing and isolate culturing therefore seems essential first steps to understand if and how these may be used to treat or prevent disease in humans and animals.

Increased levels of *E. coli*, members of the Enterobacteriaceae in the GM, have been associated with disease, such as seen in T2DM patients, as well as in acute inflammatory diseases(e.g. acute pancreatitis in humans)^42^. In rodent models of obesity and insulin resistance, increased levels of *E. coli* in the gut increased intestinal permeability and levels of circulating endotoxins leading to systemic low-grade inflammation^11,43^. In this study, fructosamine levels positively correlated with the presence of the Enterobacteriaceae in the GM. Low-grade inflammation is one of the proposed mechanisms through which gut microbiota may cause insulin resistance and T2DM. In other rodent models, host-inflammation, caused through either infection, chemical-assault or genetic predisposition, lead to increased growth of the Enterobacteriaceae family^44^, indicating that host inflammation irrespective of the cause may be the initiator of this cascade.

*Bifidobacterium* may be associated with body condition score in cats, as some of the results in this study indicated. DM cats had an increased proportion of Actinobacteria (class) compared with LN cats, which at lower taxonomic levels were seen as mostly a higher proportion of *Bifidobacterium*, and to a lesser degree to an unknown genus of the Coriobacteriaceae. In addition, the OB cats had an increased proportion of Actinobacteria compared to LN cats, and a higher proportion of *Bifidobacterium* at the genus level. The DM cats were heavier than LN cats, with a median body condition score of 6/9. A possible explanation is, therefore, that the increased proportion of *Bifidobacterium* is associated with the body condition of the cats.

*Dialister*, *Anaerotruncus* as well as some members of the Ruminococcaceae family are butyrate and/or propionate producing bacteria. A significantly lower proportion of the *Anaerotruncus* was seen in the diabetic cats in the cross sectional study, and a significantly lower proportion of *Dialister* and an unknown Ruminococcaceae genus was seen in the DM than LN cats after the dietary intervention. Decreased levels of *Anaerotruncus* have been observed in T1D children compared to controls^45^. Additionally, *Dialister* was completely absent in children who were genetically predisposed to T1D and who developed the disease later in life^46^. A recent large metagenomic study found that metformin-untreated T2DM patients had a decrease in genera containing known butyrate producers^6^. In the present study, Ruminococcaceae was found to be negatively correlated with the marker of acute inflammation SAA. Butyrate increases insulin sensitivity and energy expenditure in mice. In addition, butyrate may have immune modulating effects^9^. *In vivo*, mice fed a diet high in soluble fiber showed increased levels of interleukin 1 receptor agonist (IL-1RA) in the brain, a cytokine known to inhibit inflammatory cytokines after exposure to LPS. This effect was proposed by the authors to occur through butyrate production^47^. Butyrate may thus regulate immune responses not only in the intestine but may have a more widespread effect on the inflammatory response of the host. Therefore a lack of bacteria with the ability to produce butyrate may be an important feature of the GM of diabetic cats.

After the diet intervention, the diversity was still significantly lower in the DM cats, and this was not only true in comparison to the LN group but also in comparison to the OB group. This finding indicates that a diet intervention with a commercial high-protein diet may increase gut bacterial diversity of a healthy overweight or obese cat, but probably not for a diabetic cat. The intervention diet was a commercial diet produced to support diabetic cats by to direct the metabolism from less direct glucose absorption from dietary carbohydrate, to more controlled hepatic gluconeogenesis from amino acids. Although the diet was not formulated with the purpose of increasing gut microbial diversity, it may be that diet alone cannot increase microbial diversity in the gut of diabetic cats. Whether other measures such as probiotic supplementation or fecal transplantation alone or in combination with a diet tailored for the purpose of promoting for example butyrate producing bacteria would be beneficial should be further investigated.

Two of the predicted functional pathways that differed between the DM and LN cats were of particular interest. In the cross sectional study, there was a significant decrease in the “Ubiquinone and other terpenoid-quinone biosynthesis” pathway amongst the DM cats compared to LN. As for humans, vitamin K is an essential vitamin for cats. It is a fat soluble vitamin, which among other functions is necessary for hemostasis. Vitamin K occurs in two natural forms - phylloquinone (vitamin K1) and menaquinone (vitamin K2). Phylloquinone, is converted to menaquinone by intestinal bacteria through the “Ubiquinone and other terpenoid-quinone biosynthesis” pathway^48^. Vitamin K supplementation in elderly male T2DM patients, has been shown to increase insulin sensitivity^49^. It is possible that diabetic cats would benefit from vitamin K supplementation, but this remains to be further investigated. It should be emphasized that these results were generated using an algorithm (PICRUST) that predicts bacterial genes based on the 16S rRNA based OTU prediction. These proposed functional pathways should therefore be interpreted with caution. In addition, characterizing the gut microbiota with 16S rRNA sequencing in itself is biased due to several factors associated with amplicon gene sequencing in general^50,51^. The sequencing depth of 9000 reads used may not have been sufficient to cover more rare gut bacteria in the feline gut microbiota, and this could have potentially influenced the differences found in gut microbial diversity. However, a previous study of long-term changes in the GM in humans found that sequencing beyond a depth of 10,000 reads did not substantially increase the detection of rare species^52^, and cats as carnivores are likely to have a less diverse GM composition compared to humans^53^

One of the limitations of the study design is that, even though diet was controlled for a short period in a subset of the cats, cats were housed in different homes where several environmental factors may have differently influenced their gut microbiota. Another limitation is that the diet intervention study was conducted only in 34 of 40 Danish cats, due to cat owner preference or other considerations. Since short-term diet was excluded as a confounding factor of differences among the groups, this may have partially compensated for the smaller sample size. However, including the described diet intervention for at least a subset of the cats is still a strength, since we were able to conclude that the differences in gut microbiota detected between the groups did not seem to only be due to differences in feeding regimens. Another strength of the study was that cats from two different countries were included, since this indicates that the results may be applied to cats in general. This could be partially due to the existence of large multinational producers of pet food leading to cats being more similarly fed in different countries compared to humans. Finally, it was a strength that two control groups, both lean and obese, all within the age range of the diabetic cats were included in the present study protocol, as this indicates that the differences detected in the diabetic group do not seem to relate only to age or body composition.

In conclusion, our data indicate that the GM of diabetic cats exhibits reduced diversity compared with healthy lean as well as overweight/obese cats. The difference in diversity became more distinct compared to the overweight/obese group after a four week intervention with a high-protein/low-carbohydrate diet. Several of the bacterial genera which were decreased in the diabetic cats compared to the healthy lean cats are known to be producers of the SCFA butyrate. Serum fructosamine levels positively correlated with the Enterobacteriaceae family, known to be associated with systemic low-grade inflammation. In contrast, the Prevotellaceae family, where certain species of this family has previously been associated with improved glucose tolerance, negatively correlated with serum fructosamine levels. Finally prediction of the functional bacterial genomes indicated that the gut microbiota of diabetic cats may be less capable of Vitamin K production compared to healthy lean cats. In addition, a few novel differences were detected in the Fusobacteria, Fusobacteriaceae and *Bifidobacterium* which may relate to body condition in cats, and an unknown *Lachnospiraceae* genus and *Peptostreptococcaceae Incertae Sedis* were more frequent in diabetic cats. However, since these taxa have not previously been clearly associated with obesity or diabetes in the same or other host species their importance should be interpreted with caution.

These results should ideally be further evaluated and characterized through metagenomic shotgun-based sequencing to allow for characterization of specific bacterial species and their functional potentials that may be important for achieving improved insulin sensitivity in cats. Such initiatives might identify possible targets in the gut microbiota of diabetic cats to pursue for future treatment or prevention options of FDM, possibly serving as a model for similar treatment options of human T2DM patients.

## Methods

### Cats

In this prospective multicenter study, cats were recruited through two locations; Copenhagen, Denmark and Zurich, Switzerland. Cats were privately owned, indoor confined and more than 6 years of age. Recruitment for the Danish healthy lean, overweight/obese cats was primarily done through advertisements in local first-opinion companion animal practices, pet shops, and social media. The diabetic cats and the Swiss healthy lean, overweight/obese cats were primarily recruited from the patient population of the University Hospital for Companion Animals in Copenhagen and the Clinic for Small Animal Internal Medicine, University of Zurich, respectively. The recruitment was performed from December 2014 until December 2016. The recruited cats were categorized in three groups, the diabetic group (DM), the healthy overweight/obese group (OB) and the healthy lean group (LN). The categorization was based on the following inclusion and exclusion criteria.

All cats had to be more than 6 years of age, and not received any antibiotic treatment within the last four months, or any probiotics, or any feed additives such as prebiotics within two weeks of inclusion. Furthermore, the healthy control (LN and OB) cats were excluded if they had abnormalities above or below more than 10% of the reference interval on complete blood count, biochemistry or urine analysis, or abnormalities detected during the physical exam. In addition, the cats included in the LN and OB groups had no recent history of disease, neither evidence of chronic disease nor receiving any type of medications. The OB group cats had a body condition score^54^ of 7–9/9, while the LN cats had a body condition score of 4–5/9, at the time of inclusion. Cats with a body condition score of 6/9 were excluded to minimize the overlap in actual fat percentages between groups and to ensure a proper separation between lean and overweight/obese cats^55^.

All diabetic cats included in the study were either (i) diagnosed with diabetes mellitus on the day of inclusion based on disease history (PU/PD, weight loss, polyphagia, anorexia possibly combined with a plantigrade stance) and/or blood and urine abnormalities (blood glucose ≥14 mmol/L and glucosuria and hyperfructosemia), or (ii) had been previously diagnosed with the disease and were receiving treatment with insulin on the day of inclusion.

### Sample collection

#### Cross-sectional study

The study protocol was approved by the local ethical committee at the Department of Veterinary Clinical and Animal Sciences, University of Copenhagen, and by the Danish Animal Experimentation Inspectorate (Approval No. 2014-15-0201-00266), as well as by the Veterinary Office of the Canton Zurich, Switzerland (authorization number 23/14), and performed in accordance with the Danish and Swiss guidelines and regulations regarding animal experiments. In addition, informed consent was obtained from all cat owners. All cats included in the LN and OB group were fasted (for at least 12 h) prior to blood and urine sample collection. Blood samples were collected aseptically from the jugular vein, and a complete blood count and serum biochemistry analysis were performed. Serum from the Danish cats was also evaluated for thyroxine levels, and serum amyloid A (SAA), (Supplementary Table S2). Urine samples were collected from all the Danish cats by cystocentesis for urine analysis. The blood and urine analysis for the Danish and Swiss cats was performed in-house at the Veterinary diagnostic laboratory (University Hospital for Companion Animals, University of Copenhagen, Denmark) or at the Veterinary clinical laboratory (Vetsuisse Faculty, University of Zurich, Switzerland), respectively. Fecal samples for the GM analysis were collected by owners at home and either brought directly to the clinic on the day of inclusion (Swiss cats) or frozen at home by the owner and brought within a week after collection (Danish cats). The owners were asked to score the appearance of the fecal sample on a scale from 1–7, with 2 being considered an ideal consistency and scores >3 considered to be diarrhea (Nestle Purina Petcare, Wilkes-Barre, PA, USA). At the clinics, all fecal samples were frozen at −80 °C until further analysis.

#### Intervention study

Additional samples were collected from a subset of Danish cats (10 diabetic, 11 lean, 13 overweight/obese) after exclusively feeding a commercial high protein dry diet (Royal Canin Diabetic Feline, protein 45.2% of ME, fat 28.6% of ME, carbohydrate 26.2% of ME, crude fibre 3.8% of DM, total dietary fibre 16.0% of DM, Royal Canin, Aimargues, France) specifically formulated for DM cats for four weeks. The maintenance energy intake (MER) during the intervention was standardized with the aim to maintain body weight (BW) during the intervention by using one formula for lean and underweight cats (MER = 75 × BW^0,67^) and another for overweight/obese (MER = 130 × BW^0,4^) cats^56^. Owners of included cats were asked to take daily notes about whether the cats ate the entire portion or if any dietary discrepancies occurred.

### DNA extraction, PCR amplification and Sequencing

Fecal samples were thawed, and approximately 200 mg of each sample was extracted using the MoBio Powersoil DNA extraction kit, according to instructions from the manufacturer (MO BIO Laboratories, Inc., Carlsbad, CA, USA). After extraction, the DNA concentration was estimated using a Qubit™ 2.0 fluorometer (Thermo Fisher Scientific Inc., Waltham, MA, USA). After each PCR amplification the fragment size and quality (strengths of bands) was estimated using gel electrophoresis for all samples. The fragment size prior to sequencing was also estimated using an Agilent 4200 TapeStation™ (Agilent Inc., Santa Clara, CA, USA) for a subset of the samples. Following extraction, samples were stored at −20 °C until further analysis.The fecal bacterial microbiota composition was determined through 16S rRNA gene metabarcoding on an Illumina MiSeq platform (Illumina, San Diego, CA, USA) following^57^. In short, the V3-V4 region (341-f and 806-r primers) of the 16S rRNA gene was amplified by PCR using primers with Illumina adapter overhangs compatible with the Nextera Index Kit (Illumina). In a second PCR, dual index sequences and adapters were attached to the first PCR products and subsequently purified using Agencourt AMPure XP - PCR Purification (Beckman Coulter, Inc, Brea, California). Amplified products were quantified by Qubit® 2.0 Fluorometer and pooled equimolar prior to sequencing using PE250 sequencing chemistry.

### Bioinformatics pipeline and Statistical analysis

VSEARCH was used to merge raw paired-end Illumina sequencing reads, trim sequencing indices, adapters and primers. Merged and trimmed reads were quality filtered with maximum number of expected errors in each read of 0.5. Quality filtered reads were dereplicated and singletons were removed from the data^58^. The UPARSE algorithm in USEARCH was used to perform de novo OTU-clustering and chimera filtering^59^. Taxonomic classification was performed using LCA classifier and the SilvaMod database^60^. Subsequent bioinformatic analyses were performed using the Quantitative Insights Into Microbial Ecology (QIIME) pipeline^61^. Based on the rarefaction curves, a minimum of 9000 reads per sample was used as cut-off for further statistical analyses. PICRUSt was used to computationally predict the functional metagenome based on the 16 S gene information^62^. Prior to the PICRUST prediction the OTU clustering was repeated using a closed reference clustering and the Greengenes database in QIIME^63^.

Statistical analyses were performed using R version 3.4.1 (R Core Team, 2017). The raw GM data was rarefied, and the rarefied data was used in the subsequent statistical analyses, with the exception of alpha diversity indices. With the R package phyloseq alpha diversity (OTU richness, Chao1, Shannon index, and Simpson index) was calculated. Differences in richness and alpha diversity were statistically tested using Kruskal-Wallis two-way tests between countries (cross-sectional), and between groups (cross-sectional and intervention study). The genus level beta diversity was analyzed and plotted using principal coordinate analysis based on Bray Curtis, unweighted and weighted UniFrac dissimilarities, and tested for separation on the basis of group, sex, breed (cross-sectional and intervention) and country (cross-sectional only) using analysis of similarity (ANOSIM). Differences between groups (cross-sectional and diet intervention) and country (cross-sectional only) of specific taxa (phylum, class, order, family, and genus), as well as for the predicted functional metagenome was compared using the phyloseq and deseq. 2 packages in R. P-values for all the statistical analyses were adjusted using false discovery rate (FDR) and were considered significant when FDR < 0.05.

Bacterial genera found to have a significant influence on the variation of the first two principal coordinates of the Bray Curtis distance were determined using the envfit function in the vegan package and plotted. Canonical correspondence analysis (CCA) was used to determine interactions between selected metadata (complete blood count, biochemistry, group, country, body weight, body condition score, age, sex, neutering, and fecal score), given the microbiota dataset at OTU level.

Pairwise Spearman correlation analyses of the most abundant family level relative abundances and the body weight, body condition score, SAA, and fructosamine levels of the cats were performed. One of the DM cats had a very high SAA (60 mg/L, upper reference limit 5 mg/L), indicating acute inflammatory illness, and was found to have an overt effect on the correlation analysis, and was therefore excluded from this analysis. Pairwise Spearman correlation was used in analyses of the alpha diversity (OTU richness, Chao1, Shannon index and Simpson index) and the hematology, serum biochemistry, thyroxine and SAA levels.

## Supplementary information

Supplementary information

## Data Availability

The datasets used and/or analyzed during the current study are available from the corresponding author on reasonable request. The raw 16S rRNA gene sequencing data is available from the EMBL Nucleotide Sequence Database (ENA), with the study Accession Number PRJEB26082.
