# Supplementary material for: Diabetic cats have decreased gut microbial diversity and a lack of butyrate producing bacteria
Source: Sci Rep. 2019 Mar 18;9:4822. doi: 10.1038/s41598-019-41195-0 (PMC6423039; doi:10.1038/s41598-019-41195-0)
Supplement: Supplementary file 1 — Supplementary information [file 41598_2019_41195_MOESM1_ESM.pdf]

Diabetic cats have decreased gut microbial diversity and a lack of butyrate producing bacteria

Ida Nordang Kieler[1], Melania Osto[2], Leoni Hugentobler[2], Lara Puetz[3], M. Thomas P. Gilbert[3,4], Torben Hansen[5], Oluf Pedersen[5], Claudia E. Reusch[6], Eric Zini[6,7], Thomas A. Lutz[2], Charlotte Reinhard Bjørnvad [1]

[1] University of Copenhagen, Department of Veterinary Clinical Sciences, Dyrlaegevej 16, DK-1870 Frb. C, Denmark,

[2] University of Zurich, Institute of Veterinary Physiology, Winterthurerstrasse 260, CH-8057 Zurich, Switzerland,

[3] University of Copenhagen, Natural History Museum of Denmark, Oester Voldgade 5-7, DK-1350 Copenhagen K, Denmark,

[4] Norwegian University of Science and Technology, University Museum, 7491 Trondheim, Norway

[5] University of Copenhagen, Novo Nordisk Foundation Center for Basic Metabolic Research, Maersk Tower, Panum, Blegdamsvej 3B, DK-2200 Copenhagen N, Denmark,

[6.] University of Zurich, Clinic for Small Animal Internal Medicine, Winterthurerstrasse 260, CH-8057 Zurich, Switzerland,

[7.] University of Padova, Department of Animal Medicine, Production and Health, viale dell'Università 16, 35020 Legnaro (PD), Italy.

Table S1. Median and [range] of the age, body condition score (BCS) on a scale from 1-9, body weight (BW), and fecal score on a scale from 1-7, and number of each sex and neutering status (female entire (FE), female neutered (FN), male entire (ME) and male neutered (MN)), as well as number of each breed (Birman (BIR), Bengal (BEN), British Shorthair (BSH), Burmese (BUR), Domestic shorthair (DSH),Maine Coon (MAC), Norwegian forest cat (NOR), Ocicat (OCI), Oriental shorthair (ORI), Persian (PER), Ragdoll (RAG), Scottish Fold (SFO),Siamese (SIA), Somali (SOM), for all the cats, divided by group diabetic (DM) (n=31), healthy lean (LN) (n=34), and healthy overweight/obese (OB) (n=26).

	DM	LN	OB
Age	10.5 [6-16]	8 [6-15]	9[6-14]
BCS	6 [2-9]	5 [4-5]	7 [7-9]
BW	5.5 [3.3-8.9]	4.6 [2.6-6.9]	5.9 [4.1-8.8]
Fecal score	2[1-3]	2[1-3]	2[1-3]
Sex	7 FN, 24 MN	6 FE, 9 FN, 3 ME, 16 MN	13 FN, 13 MN
Breed	1 BUR, 24 DSH, 2 NOR, 1 OCI, 1 PER, 1 RAG, 1 SIA	7 BIR, 2 BSH, 1 BUR, 13 DSH, 1 MAC,2 NOR , 1 ORI, 2 PER, 3 SFO, 1 SIA, 1 SOM	1 BEN, 1 BSH, 23 DSH, 1 MAC

Table S2. Median and range, and number of cats (n) complete blood count, and biochemistry, including thyroxine and Serum Amyloid A (SAA) divided by group diabetic (DM), healthy lean (LN), and healthy overweight/obese (OB)

		DM				LN				OB			
	Laboratory reference values	n	median	min	max	n	median	min	max	n	median	min	max
White blood cell count ($x10^9$/L)	4.1-19.5	29	11.09	4.21	23.03	32	8.55	3.69	24.1	27	5.53	2.7	13.16
Immature Neutrophils ($x10^9$/L)	0-0.3	29	0	0	0.23	32	0	0	1.09	27	0	0	0
Neutrophils ($x10^9$/L)	2.09-12.2	29	6.76	2.58	18.68	32	4.55	0	20.5	27	3.52	1.12	9.22
Lymphocytes ($x10^9$/L)	0.95-6.6	29	2.8	0.94	5.24	32	2.65	0.84	5.89	27	1.45	0.53	2.73
Monocytes ($x10^9$/L)	0-0.75	29	0.18	0	0.78	32	0.22	0	0.46	27	0.14	0.05	0.38
Eosinophils ($x10^9$/L)	0-3	29	0.74	0.06	1.5	32	0.53	0.03	2.5	27	0.23	0.05	2.69
Basophils ($x10^9$/L)	0-0.3	29	0.01	0	0.25	32	0.01	0	0.09	27	0	0	0.3
Erythrocytes ($x10^{12}$/L)	6.3-11.77	29	9.43	6.88	11.03	32	9.285	6.76	11.34	27	9.08	7.36	11.51
Hemoglobin (g/dL)	5-17	29	7.7	6.3	16.7	32	8.7	5.9	15.9	27	8.2	6.5	14.4
Packed Cell Volume (L/L)	0.29-0.5	29	0.383	0.315	0.48	32	0.401	0.283	0.49	27	0.394	0.304	0.46
Thrombocytes	190-430	28	304.5	72	661	26	195.5	13	317	26	218	26	431

$(x10^9 /L)$													
Alanine aminotransferase (U/L)	6-84	30	59	23	123	32	54	36	82	27	53	29	83
Alkaline phosphatase (U/L)	11.2-96	30	114	22	144	32	48	11	95	27	63	28	94
Gamma-glutamyltransferase (U/L)	0-5	23	0	0	2	21	0	0	3	26	0	0	1
Glucose (mmol/L)	3.6-10.9	30	21.2	6.8	35.5	32	6	3.1	12.9	27	7.6	2.4	12
Blood urea nitrogen (mmol/L)	6.3-19.9	30	12.2	7.26	28.84	32	9.515	6.5	19.2	27	9.44	6.9	11.3
Creatinine (umol/L)	60-182	30	94	52	212	32	134	93	174	27	132	87	182
Cholesterol (mmol/L)	2.3-7.5	30	6.325	2.79	12.59	32	4.9	1.97	6.8	27	4.43	2.13	8.8
Bilirubin (umol/L)	0-3.85	28	0.7	0.1	2.5	32	0.8	0.1	2.5	27	0.8	0	2.2
Albumin (g/L)	27-44	30	36.24	29.61	42.81	32	36.64	29.46	43.38	27	37.2	30.96	41.58
Total protein (g/L)	58-88	30	73.52	64.7	89	32	72.055	65	88	27	71.43	65.63	78.62
Globulins (g/L)	28-53	30	36.9	29.95	53	32	34.87	28.67	53	27	34.36	30.76	39.46
Fructosamine (umol/L)	182-430	30	623	360	1233	32	322	242	430	27	355	287	414

Bile acid (umol/L)	0-5	23	2	0	10	21	1	0	4	26	1	0	5
Calcium (mmol/L)	1.95-3.8	30	2.415	1.98	2.84	32	2.49	1.95	3.51	27	2.54	2.15	3.33
Magnesium (mmol/L)	0.7-1.5	23	0.92	0.67	1.21	21	0.9	0.72	1.14	26	0.97	0.84	1.06
Phosphorous (mmol/L)	0.6-1.98	30	1.36	1.2	2.62	32	1.31	0.71	1.84	27	1.15	0.96	1.6
Sodium (mmol/L)	142.2-181.5	30	150.6	142	164	32	153.35	147.4	166	27	152.7	146.9	161
Potassium (mmol/L)	3.42-5.94	30	4.815	3.8	5.61	32	4.425	3.64	5.5	27	4.21	3.64	5.6
Chloride (mmol/L)	108.9-144.1	30	115.5	103	140.8	31	120	111	133.04	27	118.9	114.78	133.6
SAA (mg/L)	0-5	23	0.5	0	64.4	21	0	0	1.1	26	0.6	0	1.3
Thyroxine (nmol/L)	10-46.4	23	18.9	6.44	35.6	20	29.75	19.9	46.2	26	35.15	28.1	43.5

Table S3. Differences in rarefied relative abundance of the most common predicted pathways, between diabetic (DM), (n=23)and lean (LN), (n=24) cats as well as overweight/obese (OB), (n=15) compared to lean cats, for the cross sectional, as well as between DM (n=11)and LN (n=12) cats and OB (n=13) after a four week diet intervention with a commercial high-protein diet. Differences are illustrated as effect size (E) expressed as the difference between groups in terms of the binary logarithm (2^{n}), including the standard error (SE) of the effect size and the false discovery rate (FDR) adjusted p-value.

		Predicted pathway	E	SE	FDR
Cross-sectional					
	DM vs. LN	Ubiquinone and other terpenoid-quinone biosynthesis	-1.1	0.3	0.03
	OB vs. LN	Ubiquitin system	3.1	0.8	0.01
Intervention					
	DM vs. LN	Lipoic acid metabolism	1.5	0.5	0.01
		Staphylococcus aureus infection	1.7	0.5	0.01
		Transcription related proteins	1.6	0.5	0.01
	OB vs.LN	Staphylococcus aureus infection	1.7	0.5	0.02

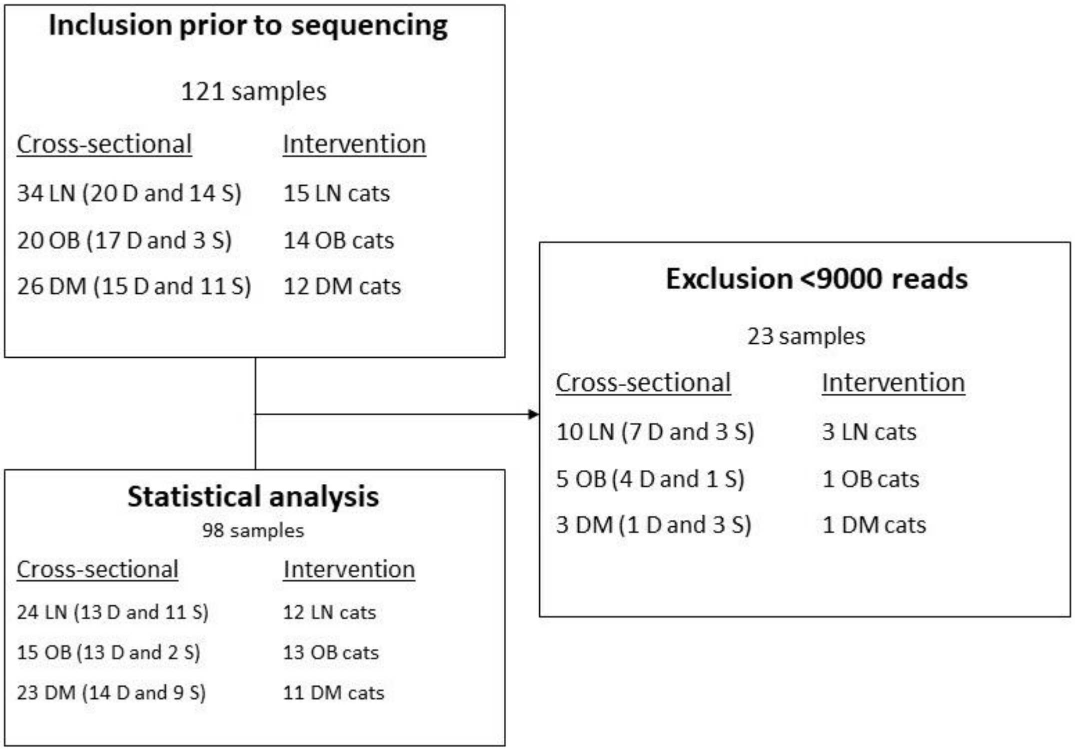

Figure S1. Flowchart illustrating which sample were excluded on the basis of having less than 9000 reads, and from what group of cats, lean (LN), overweight/obese (OB) or diabetic (DM) they belonged to, and if they were Danish (D) or Swiss (S)

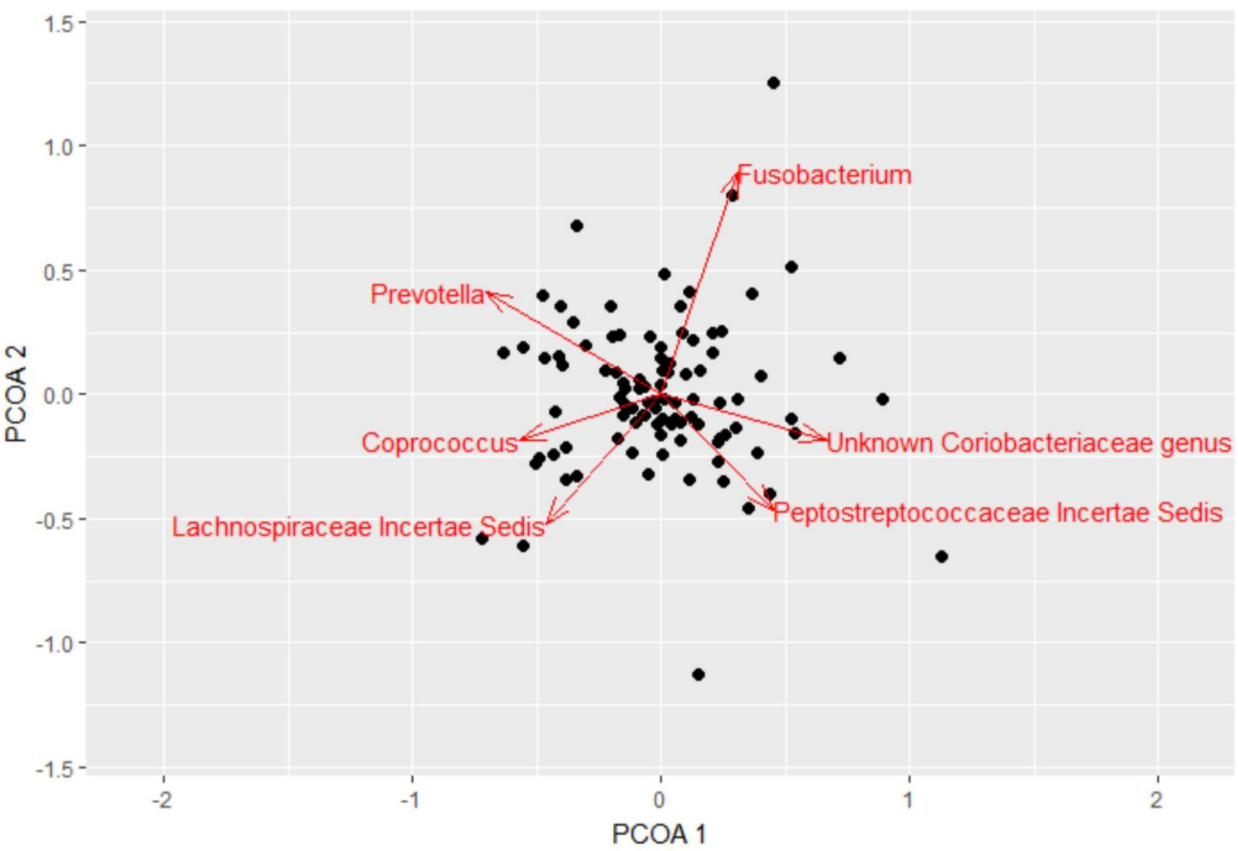

Figure S2. Bacterial genera found to have a significant influence on the variation of the first two principal coordinates (PCOA1 and PCOA2, respectively) of the Bray Curtis dissimilarity at OTU level for all the cats (n=62, from the three groups diabetic (n=23), overweight/obese (n=15), and lean (n=24)) in the cross sectional study.

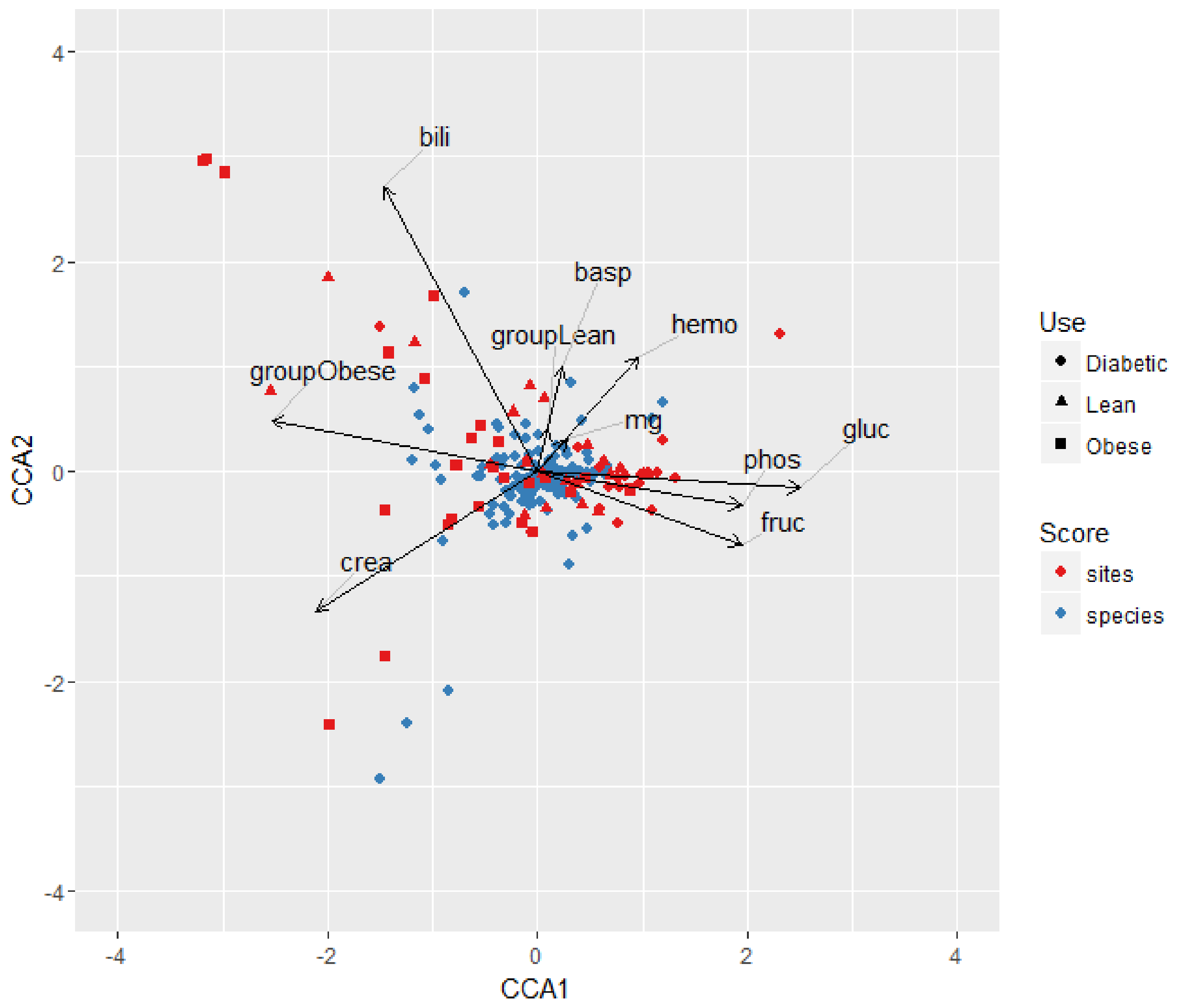

Figure S3. Canonical correspondence analysis (CCA) was used to determine interactions between selected metadata (complete blood count, biochemistry, group, country, body weight, body condition score (BCS), age, sex, neutering, and fecal score) of the diabetic, healthy lean, overweight/obese cats, given the microbiota dataset at OTU level. Metadata variables found to have a significant (adjusted p-value, FDR <0.05) influence on the OTU level gut microbiota variation (from all the cats (n=62, from the three groups diabetic (n=23), overweight/obese (n=15), and lean (n=24)) in the cross sectional study), significant factors was plotted against the first (CCA1) and second (CCA2) components.

groupObese=Overweight/obese group, groupLean=Lean group, bili=Bilirubin, crea=Creatinine, basp=Alkaline phosphatase, hemo=Hemoglobin, mg= Magnesium, phos= Phosphorous, gluc=Glucose, fruc= Fructosamine

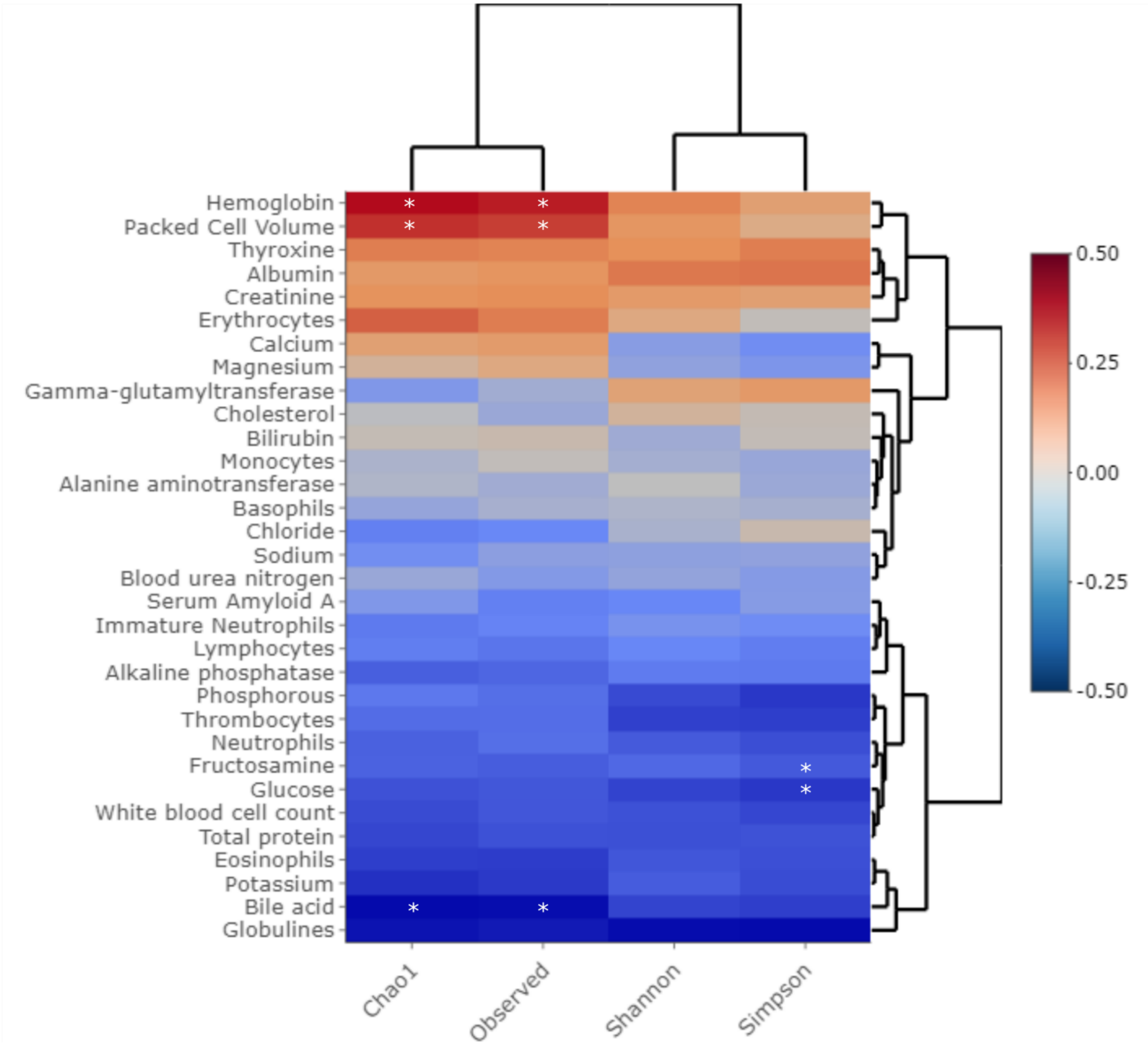

Figure S4. Heatmap of the correlation between the alpha diversity and hematology, serum biochemistry, Serum Amyloid A (SAA) and thyroxine levels. Pairwise Spearman correlation of the observed richness (Observed), Chao 1, Shannon diversity and Simpson diversity and hematology, serum biochemistry, Serum Amyloid A (SAA) and thyroxine levels of all the cats (diabetic (n=23), lean (n=24), and overweight/obese (n=15)) from the cross-sectional study was performed. P-values were adjusted using FDR and were considered significant when FDR<0.05. *=FDR<0.05
